# Non‐Invasive Temporal Interference Electrical Stimulation Modulates Neurotransmitter Release and Improves Aberrant Neural Oscillations in Alzheimer's Disease

**DOI:** 10.1002/cns.70848

**Published:** 2026-05-05

**Authors:** Linyan Wu, Sinan Li, Liang Huang, Long Li, Ping Zhou, Zhiyuan Lu, Tian Liu, Jue Wang

**Affiliations:** ^1^ School of Rehabilitation Sciences and Engineering University of Health and Rehabilitation Sciences Qingdao China; ^2^ The Key Laboratory of Biomedical Information Engineering of Ministry of Education, Institute of Health and Rehabilitation Science, School of Life Science and Technology Xi'an Jiaotong University Xi'an Shaanxi P. R. China; ^3^ The Key Laboratory of Neuro‐Informatics & Rehabilitation Engineering of Ministry of Civil Affairs Xi'an Shaanxi P. R. China

**Keywords:** Alzheimer, neuronal oscillatory rhythms, neurotransmitter expression, temporal interference

## Abstract

**Background:**

Modulating brain oscillations has significant therapeutic promise. Traditional non‐invasive neuromodulation techniques can alleviate clinical signs of Alzheimer's disease (AD) by restoring normal neural oscillatory activity in certain brain regions. As a novel non‐invasive brain modulation technique, temporal interference (TI) has been demonstrated to precisely control hippocampus neural oscillations while minimizing its impact on cortical neural activity, but its exact mechanism of action is still unclear.

**Method:**

We simulated and experimentally measured the intracranial electric field under TI to determine the precision of TI intervention. Subsequently, TI stimulation was applied to the APP/PS1 transgenic AD mouse model, and the impact of TI stimulation on the stimulated brain region was compared from the perspectives of behavior, electrophysiology, and cell biology.

**Results:**

This work showed that in the APP/PS1 Alzheimer's disease mice model, TI stimulation significantly increased GABA levels and decreased NMDA receptor activation at the targeted region. Following neurotransmitter regulation, the rhythm of the gamma oscillations they associate also changed. This, in turn, influenced other memory‐related neural oscillation frequencies and brain regions through cross‐frequency coupling and brain connectivity, ultimately improving the behavioral performance of AD model mice.

**Conclusions:**

The results of our work demonstrated how TI stimulation alters brain oscillations to enhance memory in mice with Alzheimer's disease, offering a possible theoretical foundation for TI's clinical application.

## Graphical abstract

Gamma frequency band temporal interference (TI) electrical stimulation targeting hippocampus can enhance hippocampal neuronal activity through the regulation of GABA and NMDA receptor expression. Along with the recovery of abnormal endogenous brain oscillations in neuronal activity, gamma band TI improved the memory function of AD model mice.

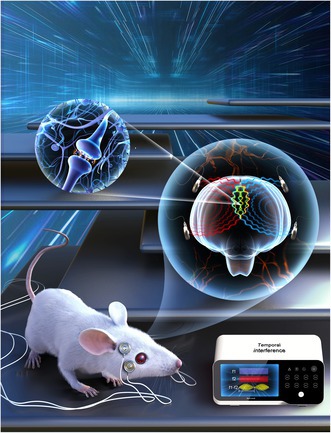

## 1 Background

The brain exhibits inherent or preferred oscillations in response to specific stimulus frequencies, and Alzheimer's disease may be the result of disruptions to these biased intrinsic oscillations [1, 2]. The low power spectra of gamma band LFP and the steadily declining coupling between gamma frequency bands and other frequency bands are thought to be the possible neurophysiological indicators of AD cognitive impairment [3, 4]. So if specific transcranial stimulation techniques can be utilized to encourage or restore rhythmic neural activity within the gamma frequency range, it is thought to improve cognitive function in Alzheimer's patients [5]. Research has demonstrated that an imbalance between excitation and inhibition in the brain, either through overactivity of the excitatory GABAergic system or dysfunction of the inhibitory GABAergic system, is responsible for the disturbance of endogenous neural oscillations in AD patients [6, 7, 8]. GABA‐B receptors bind to G proteins and regulate the release of excitatory and inhibitory neurotransmitters in the hippocampus, thalamus, spinal cord, and other brain regions [9]. According to certain research, early GABAergic system disruption may cause memory and cognitive issues in Alzheimer's patients. The lack of GABA‐B receptors causes aberrant connections between pyramidal cells and PV GABAergic interneurons, which shows up as aberrant gamma oscillatory rhythm activity in hippocampal neurons [7, 10]. Furthermore, the GluN1 subunit, a component of NMDA receptors, is the most prevalent receptor in PV interneurons. Defects in PV interneuron activity, which may be connected to the neuronal gamma oscillation cycle, can also result from deviation from this subunit [11]. Meanwhile, research has demonstrated that excitotoxicity brought on by excessive activation of NMDA receptors is also linked to neuronal death in the brain afflicted by Alzheimer's disease [12, 13]. Based on the above viewpoint, GABA‐B and GluN1 receptors can function as physiological biomarkers to investigate the neuronal regulatory mechanisms of gamma frequency treatments on Alzheimer's disease.

**FIGURE 1:**
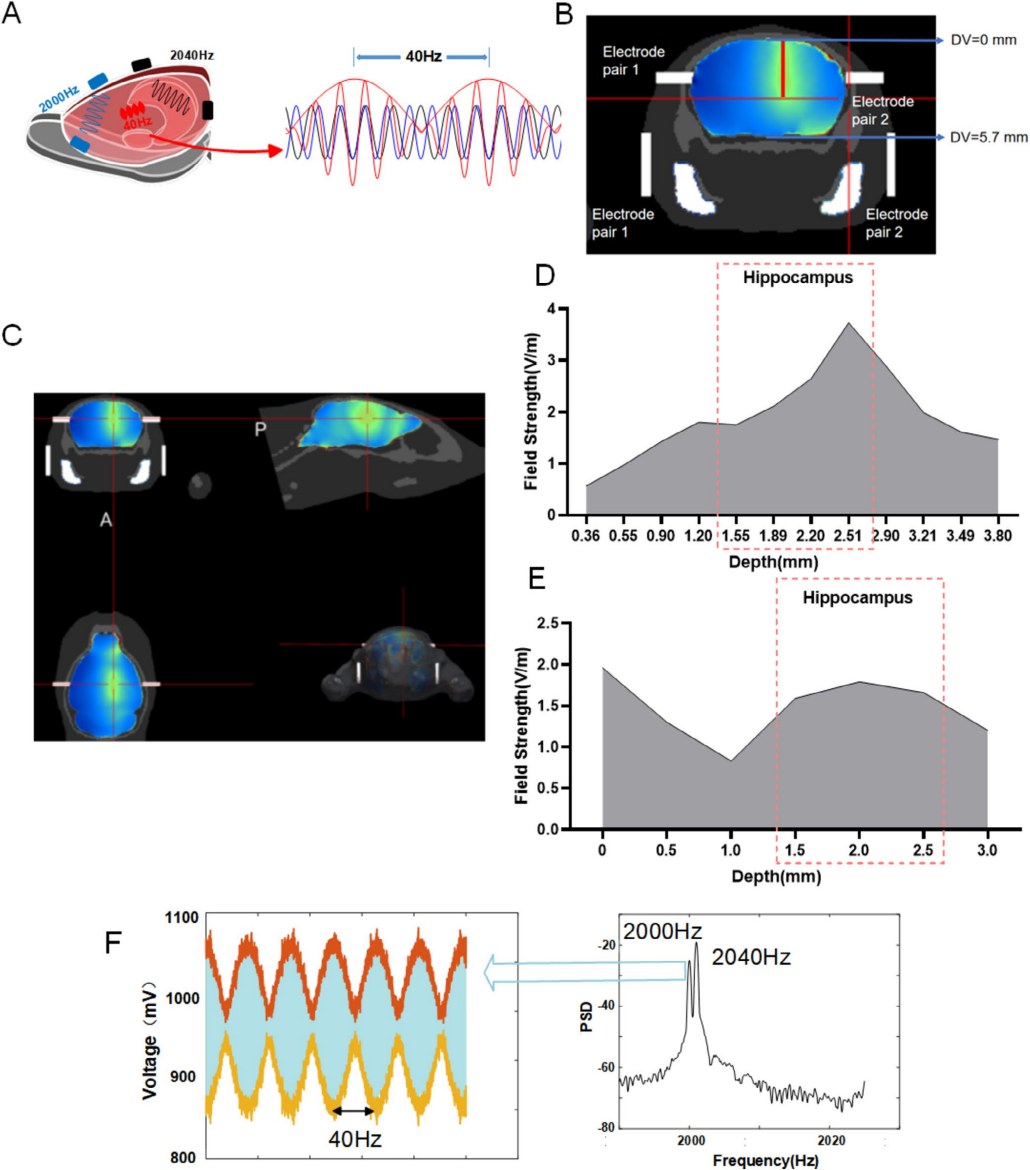
Fundamentals and simulation validation of TI hippocampal stimulation. (A) Schematic of TI technology, coupling high‐frequency stimulation at 2000 Hz and 2040 Hz to generate low‐frequency stimulation at 40 Hz within the brain. (B) Schematic of the simulation model. (C) Simulation schematic targeting the right hippocampus with TI. (D) Simulation of electric field intensity at different depths from cortex to hippocampus. (E) Measured results of electric field strength at different depths from cortex to the hippocampus. (F) The signal acquired at the location marked by the red box in Figure E. Power spectral analysis reveals peaks at 2000 Hz and 2040 Hz, indicating stimulation at the hippocampal region. In the time domain, a 40 Hz envelope can be observed, resulting from the coupling of two high‐frequency signals.

The hippocampus, which is situated much below the cortex, is a common target for neural regulation of AD. Currently, the hippocampus is the focus of numerous non‐invasive neural control methods used to treat Alzheimer's. It is challenging to identify which brain regions are stimulated to enhance cognitive function when using transcranial electrical or transcranial magnetic techniques, which may also affect the study of regulatory intervention mechanisms. Since its proposal, temporal interference, a unique non‐invasive brain modulation approach, has generated a lot of interest since it can produce accurate and non‐invasive focusing while reducing the intervention effect on the cortex during deep brain intervention. The viability of TI stimulation targeting brain regions like the hippocampus and prefrontal cortex has been demonstrated by simulation studies [14, 15]. Grossman tried to use TI to treat Alzheimer's patients after proposing it and confirmed its beneficial effects on cognitive function [16, 17]. Additionally, TI has shown promise in clinical trials for conditions like sleep problems and motor dysfunction [18, 19]. However, TI technology's intervention process might differ from other non‐invasive electrical stimulation methods like tDCS and tACS because it accomplishes focused intervention by superimposing two high‐frequency signals to create a stimulus envelope (Figure 1A). Therefore, even though TI has demonstrated promise in clinical trials intended to improve cognitive capacities in individuals with Alzheimer's disease, further research on its intervention mechanisms and the therapeutic mechanisms of TI on cognitive function is still required.

We put forward our hypothesis that TI stimulation in the gamma frequency band can enhance hippocampal neuronal activity through the regulation of neurotransmitter dynamics, thereby restoring abnormal endogenous brain oscillations in AD and improving memory function in AD model mice. This hypothesis builds upon the proven effectiveness of TI methods in activating and modulating hippocampal activity. We first used a simulation model to perform simulation research in order to determine the proper stimulate parameters for TI stimulation. We recorded neuronal electrical pulses that stimulate targets while concentrating on neurotransmitters like GABA and NMDA in animal research. We investigated variations in LFP in the frequency band, focusing on power spectrum and cross‐frequency coupling, by integrating immunofluorescence labeling of neurotransmitters. This allowed us to provide a mechanism explanation for the modulation of endogenous oscillations in the brain. Since the transmission of some neurotransmitters between the prefrontal cortex and the hippocampus had an advantageous direction, we designed an experiment to compare whether TI targeting the hippocampus and targeting the prefrontal cortex had different biochemical indicators in order to confirm that the mechanism explanation obtained from the research results was due to TI technology stimulating the hippocampus rather than intervening in other cognitive‐related cortical regions (prefrontal cortex) during the process of stimulating the hippocampus. By comparing the results of these biomarkers with behavioral outcomes, we intend to show that TI stimulation targeting the hippocampus can enhance memory performance in AD.

## 2 Materials and Methods

### 2.1 TI Electric Field Simulation

The Computational Geometry Algorithm Library (CGAL) was utilized in this study to split the model's finite element mesh and perform electric field modeling calculations using an open‐source mouse model [20, 21]. The electric field in the brain was then computed using the finite element solver GetDP [22]. We can mimic the effects of TI stimulation by superimposing the intervention results of two channels using different frequency current stimuli.

Using simulation, we initially investigated the hippocampus‐targeting TI's stimulation parameters. The right hippocampus was the target of two pairs of electrodes applied to the scalp. Evidence that the right hippocampus was prominent in spatial memory [23], which was compromised in AD, led to its selection. In order to separate a variety of tissue types using high‐resolution magnetic resonance imaging (MRI) and slice data, the electric field distribution was computed in Sim4Life Virtual Zoo model [24]. By employing finite element analysis, the mice were separated into 4.18 million voxels. Simulations with TI settings that target the hippocampus were then carried out. The voltage difference obtained by simulating various positions was divided by the distance between positions to get the envelope strength. We also simulated the prefrontal cortex's outcomes using the same technique.

### 2.2 Experimental Animals

9‐month‐old male APP/PS1 mice were used in this investigation, and all tests were conducted in accordance with protocols authorized by the Animal Protection and Use Committee of Xi'an Jiaotong University. The animals were housed in a facility with a 12‐h light–dark cycle, a temperature‐controlled environment (22°C ± 0.5°C), and unrestricted access to food and water. Every effort had been made to reduce the number of animals used and the amount of misery they endure. APP/PS1 transgenic mice strain C57/BL6‐Tg (Hu APP695swe, PSEN1‐d E9) were bought from the Institute of Medical Experimental Animals, Chinese Academy of Medical Sciences. These mice were divided into 6 groups, with 10 animals in each group: (1) AD group, (2) Sham group, (3) 135 μA TI group, (4) 750 μA TI group, (5) TI HPC (hippocampus) group, (6) TI PFC (prefrontal cortex) group. We did not design a control between normal mice and AD mice because the relevant research had been published in previous articles [25].

### 2.3 TI Stimulation in APP/PS1 Mice

We employed a constant current stimulator (NervioX‐H0800 of NeuroDome Company) to generate TI stimulus waveforms, utilizing reverse‐phase current drive technology to prevent current crosstalk. In our experiments, we used two pairs of electrodes from the stimulator, which generated sine waves with frequencies of 2000 Hz and 2040 Hz. Choosing 40 Hz for stimulation was because this frequency band had been proven to have a positive effect on AD treatment [26]. The anode of TI stimulation was a silver wire with a diameter of 1 mm, which was wrapped with insulating material on the outside and had an exposure length of 4 mm. The cathode was a medical‐grade hydrogel electrode with a diameter of 5 mm. Using finite element simulation, the stimulation electrode was positioned in accordance with the ideal electrode position. The sham stimulation group switched to a sham stimulation mode, which linked the stimulator without stimulation at other times and only provided 3 s of stimulation at the start and finish of the stimulation; 135 μA and 750 μA currents were evaluated in an initial experiment; 750 μA was chosen for further studies and split into TI HPC and TI PFC groups.

### 2.4 Electrode Placement

In “Verification of hippocampal targeting” section, we performed craniotomy and implanted collection electrodes in the hippocampus of mice (AP = −1.94 mm, ML = 1.25 mm), implanted them from the cortex downwards, and recorded the collected electrical signals every 0.5 mm to calculate the electric field strength generated by TI in the skull.

The collecting electrodes for the APP/PS1 animals were implanted soon after the TI stimulation phase in the tests that followed. A 2% (v/v) concentration of isoflurane oxygen was used to anesthetize mice. After that, the mice were put on a thermostat‐controlled heating pad. The amount of anesthetic was increased as necessary, and the depth of anesthesia in the mice was monitored in real time. The head's hair was cleaned and shaved. Following a craniotomy performed just above the hippocampus, the LFP acquisition electrode was placed. As a reference point for the acquisition electrode, screws were placed above the cerebellum. After implantation, the electrode was surrounded with copper mesh and secured with dental base acrylic glue to minimize external electromagnetic interference. The mice were evaluated twice daily for the next two days, and if they displayed any symptoms of pain or stress, they were administered carprofen (2 mg/kg). Two days following electrode implantation, behavioral assessments were administered to the animals.

### 2.5 Y‐Maze Test

We spent two days acquainting the mice with the Y‐maze environment. The formal experiment to evaluate the mice's learning capacity in the Y maze then started. Throughout the experiment, the mice were positioned in either arm of the Y maze. For two tests in a row, mice must enter separate arms. The mouse must enter arm C rather than arm B the second time if it began at arm A and made its first foray into arm B. If they chose incorrectly, they would be punished with a 2 s 15 V electrical stimulation. An example of one trial was the process outlined above. Each group in our experiment had 40 trials, with each trial consisting of two tests. To confirm the quality and consistency of the collected LFP data, the full experiment was completed in a single day.

Our goal was to determine whether stimulation at the target site may alter hippocampus neuronal activity when TI stimulation was shown to be specifically targeted towards the hippocampus. In order to evaluate LFP in the hippocampus, we performed TI stimulation on 30 ad mice (10 in the control group, 10 in the sham group, and 10 in the 750 μA TI group) for 21 days in a row. After 20 min of stimulation each day, recording electrodes were implanted. The animals' memory function was known to be reflected in the Y maze [27]. Therefore, we conducted the Y maze experiment on all mouse groups in order to better contrast the effect of TI stimulation on hippocampus activity. We then recorded the accuracy of the mice's activities in the Y maze, which was defined as the number of times the mice explored different arms in the Y maze consecutively/total exploration times [28]. TI current ratio 1:1 (2.04 kHz, 0.75 mA, and 2 kHz, 0.75 mA) and sham group (2.04 kHz, 0.75 mA, and 2 kHz, 0.75 mA, lasting 3 s at the start and finish) were the electrode configurations used for the stimulation. Since there was evidence that hippocampus gamma band oscillations play a role in episodic memory, we chose the gamma band difference‐frequency at 40 Hz [29].

### 2.6 Electrophysiological Analysis

All of the analyses were carried out in MATLAB using built‐in and custom‐written functions as shown in the following sections. The mouse LFPs were recorded using a 64‐channel data acquisition system (Cerebus, Blackrock Microsystems, Salt Lake City, USA). LFPs were captured at a 10 kHz sampling rate, amplified (×300), and bandpass filtered (discrete Fourier transform filter, 0.3–250 Hz). Since the animal made a decision before entering the center of the maze, we chose the time when the mice's hind limbs left the chosen area as the end time, and 2 s before this time as the time to be analyzed. We chose the local field potentials in the choice area (Triangle area) of the Y‐maze as the neural electrophysiological activity during the mice's engagement in memory tasks.

### 2.7 Spectral Analyses, Filter Settings, and Amplitude and Phase Time Series Extraction

The Cerebus NeuroExplorer application was used to estimate the power spectrum of the electrophysiological data of the mice in the Y‐maze selection area with a window size of 0.1 s and a step size of 0.05 s. The experiment's electrophysiological data was then selected during a time frame devoid of low‐frequency interference and power frequency noise. 30–45 Hz filters were used to filter the gamma band, and 4–7 Hz filters were used to filter the theta band. The power spectra density was computed using the MATLAB toolbox Chronux (http://chronux.org) using multi‐taper spectral estimation. We calculated the power spectrum ratio between the target frequency band and the adjacent frequency band (as the background frequency band) to verify that the power spectrum changes after TI stimulation were not affected by background spectrum activity. The average power spectrum estimation result was obtained by averaging all power spectrum data across the entire time period.

### 2.8 The MI and the Phase‐Amplitude Comodulogram

The modulation index (MI) measured the strength of the theta‐gamma phase‐amplitude connection. The local field potential was filtered twice separately to provide a slow (theta, 4–7 Hz) and a fast (gamma, 30–45 Hz) frequency range of interest. Phase (theta) and amplitude (gamma) time series were then obtained using theta and gamma filtered signals, respectively. A composite time series consisting of instantaneous phase vs. amplitude values was used to calculate the mean amplitude of each phase bin. The MI was then used to measure the strength of the phase‐amplitude coupling between the gamma amplitude and theta phase.

### 2.9 Granger Causality Connection Method

In order to quantify statistical dependencies and further explore the causal relationships between HPC and PFC as well as the direction of information flow between these structures, we calculated Granger causality in the frequency domain. We filtered the local field potential signal, then used thresholding to remove artifacts, and conducted stationarity tests on the data using the “adftest” function in MATLAB to ensure that the data met the assumptions of Granger causality analysis. We examined our data using multivariate Granger causality (MVGC). In order to determine the ideal model order, the first step was to estimate the model order using the Akaike information criterion (AIC). The vector autoregression (VAR) model was then estimated using the chosen model order and the Levinson Wiggins Robinson (LWR) algorithm. The VAR model then generated the autocovariance sequence. Lastly, partial Granger causality was calculated in the frequency domain using the autocovariance sequence.

## 3 Pathology and Immunofluorescence Analysis

### 3.1 Brain Tissue Fixation and Sectioning

Euthanasia mice were perfused and preserved with frozen 0.1 MPBS solution and 4% PFA. Mice were anesthetized with pentobarbital sodium (50 mg/kg, intraperitoneal injection) prior to perfusion. Anesthesia depth was confirmed by absence of paw withdrawal reflex before perfusion with 0.1 MPBS followed by 4% PFA. The brain was then dissected and incubated for 3 days in a 4% PFA solution at 4°C before being transferred to a 30% (v/v) sucrose solution at 4°C for a further 3 days of incubation. The brain was frozen with dry ice after this time of cryopreservation, and coronal sections were made every 40 μm with a frozen slicer.

### 3.2 Immunofluorescence

The brain slices were fixed for 30 min in 4% PFA and then infiltrated for 30 min with 0.1% Triton X‐100 (Sigma‐Aldrich). The nonspecific protein was sealed for 1 h at room temperature with 3% BSA in 0.1 MPBS solution, then incubated with anti‐rabbit antibodies: GABA‐B (1:100, ab238130, Abcam), GluN1 (1:200, ab193310, Abcam), Aβ (1:200, 13075SS, Novus) at 4°C overnight. After rinsing with PBS, slices were transferred to a 3% BSA‐PBS solution containing goat anti‐mouse (Alex Fluor 594, 1:1000, A11005, Invitrogen) and anti‐rabbit (Alex Fluor 488, 1:1000, A‐11034, Invitrogen) secondary antibodies. 4′,6‐Diamidino‐2‐phenylindole (DAPI) was used to mark the nuclei. High‐resolution digital pathological scanning system (3D HISTECH Pannoramic MID II with ×20, ×40 and ×63 objectives) was used to detect fluorescence.

## 4 Statistics

### 4.1 LFP Analysis

Univariate ANOVA was used to examine the Granger Causality Analysis (GCA), MI, and Gamma band power spectrum between groups. After confirming that LFP data follow a normal distribution, we used Levene variance homogeneity test to determine the method of post comparison. The power spectrum data were examined with the LSD hypothesis equivariance test after the homogeneity test; GCA and the MI data were tested with the Tamheni T2 non‐hypothesis equivariance post‐test. In these studies, a confidence level of *p* < 0.05 was employed.

### 4.2 Quantification of Immunopositive Cells

Immunofluorescence quantifications were performed with CaseViewer software and ImageProPlus software. With a ×80 objective lens, GABA‐B and GluN1 were quantified in each region. We chose three perspectives with a total size of 800 × 300 μm^2^ for each section's cell count. With a ×63 objective lens, Aβ was quantified in each region. We calculated the average area of individual Aβ deposits within the field of view. The average value and standard deviation of the number of cells in each group were calculated. After confirming that the data conformed to the assumption of normal distribution and homogeneity of variance, ANOVA analysis with LSD hypothesis was used to test the data. The alpha level of type I error was set to 0.05 in order to reject the erroneous hypothesis. The information was presented as mean standard deviation (SEM). One‐way ANOVA was used to compare the number of neurogenic cells stained by GABA‐B, NMDA, and Aβ in each group.

## 5 Results

### 5.1 TI Focus Targeting Effect Verification

We used the right hippocampus of mice as the target of TI stimulation and statistically examined the electric field strength of TI. The simulation results show that TI stimulation at frequencies of 2000 Hz and 2040 Hz could produce focus on the mouse hippocampus (Figure 1C). Different deep field strengths from the cortex downwards on the stimulation target were shown in Figure 1D. Using intracranial electrodes implanted in the right hippocampus to measure the potential field strength to the long axis of the hippocampus, we stimulated mice with the same TI parameters. As can be seen from the actual test results, with the envelope field strength in the hippocampus region reaching 1.9 V/m, nearly twice the field strength of the non‐target area (like DV 1 mm) (Figure 1E), the measured electric field strength trend results were in agreement with our simulation model. Figure 1F shows the signal used for analysis.

### 5.2 Gamma TI Stimulation Improves Cognitive and Behavioral Performance in AD Mice

**FIGURE 2:**
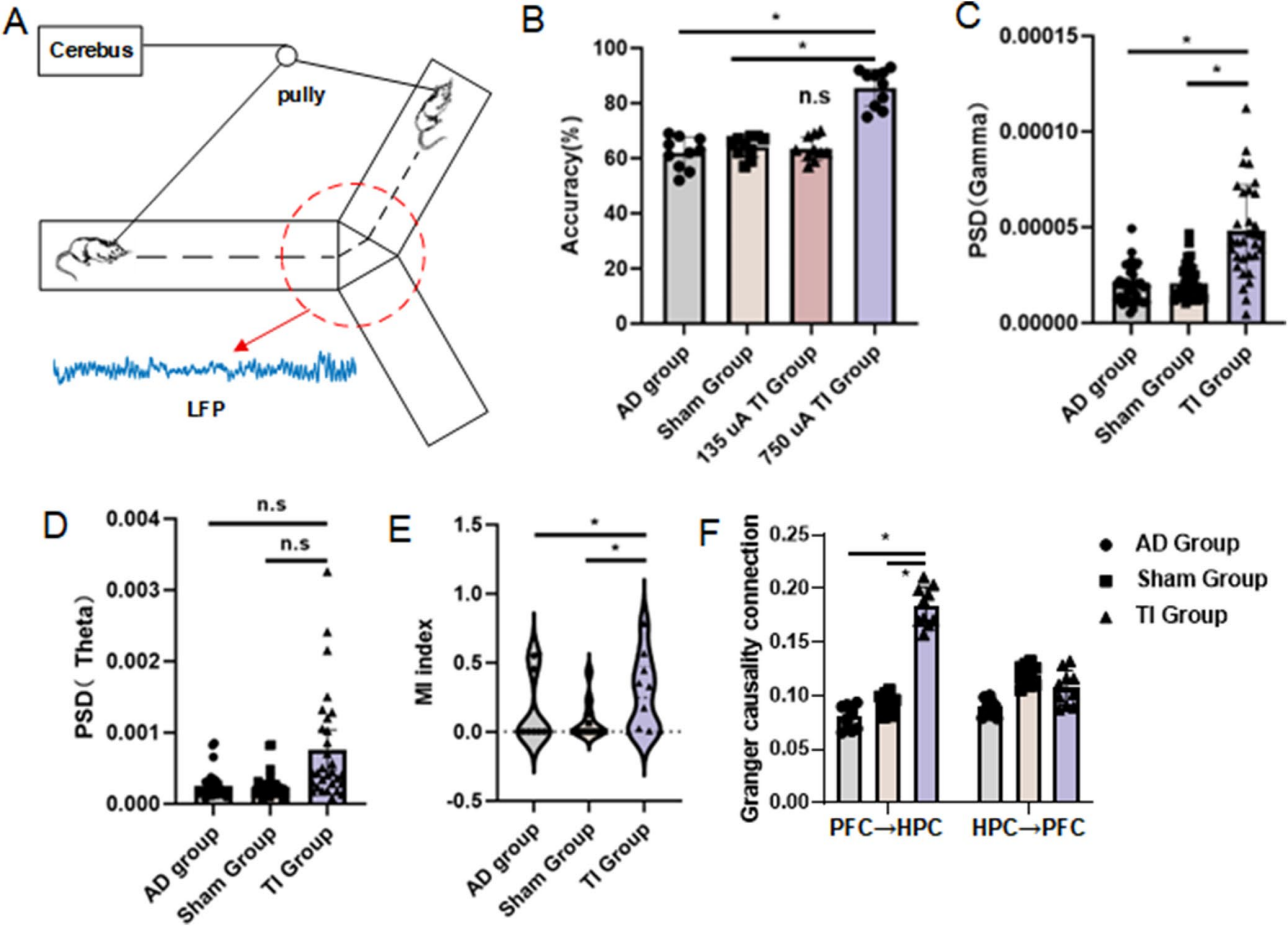
Behavioral experiments and local field potential signals in the hippocampal region (TI group is the abbreviation for 750 μA TI group, *p < 0.05, n.s.: Not significant). (A) Behavioral paradigms. (B) Behavioral results. (C) Power spectra in the gamma band of the hippocampus. (D) Power spectra in the theta band of the hippocampus. (E) Cross‐frequency coupling between gamma and theta bands in the hippocampus. (F) Granger causality connection from hippocampus to prefrontal cortex and from prefrontal cortex to hippocampus.

After determining the TI parameters through simulation, we evaluated how well the mice behaved in the Y maze test. The Y‐maze paradigm is shown in Figure 2A. The correct selection rate of the control group mice in the Y maze was 61.2% ± 5.7%, the correct selection rate of the sham stimulation group was 63.3% ± 3.8%, and the correct selection rate of the 135 μA TI stimulation group was 62.8% ± 4.3%. Our experiments with a 135 μA TI had no significant change in the behavior of mice, despite simulations showing focused activation in the mouse brain's hippocampus. Considering that some articles had conducted higher intensity stimuli [30], the stimulation current was increased to 750 μA, and behavioral verification was completed. After regulating the hippocampus with 750 μA TI stimulation, the correct selection rate of AD mice in the Y maze was 84% ± 6.7% (*p* < 0.05, Figure 2B). Therefore, a current of 750 μA for TI stimulation (TI group) was used in the subsequent work.

### 5.3 Gamma TI Stimulation Improves Neural Activity in AD Mice

**FIGURE 3:**
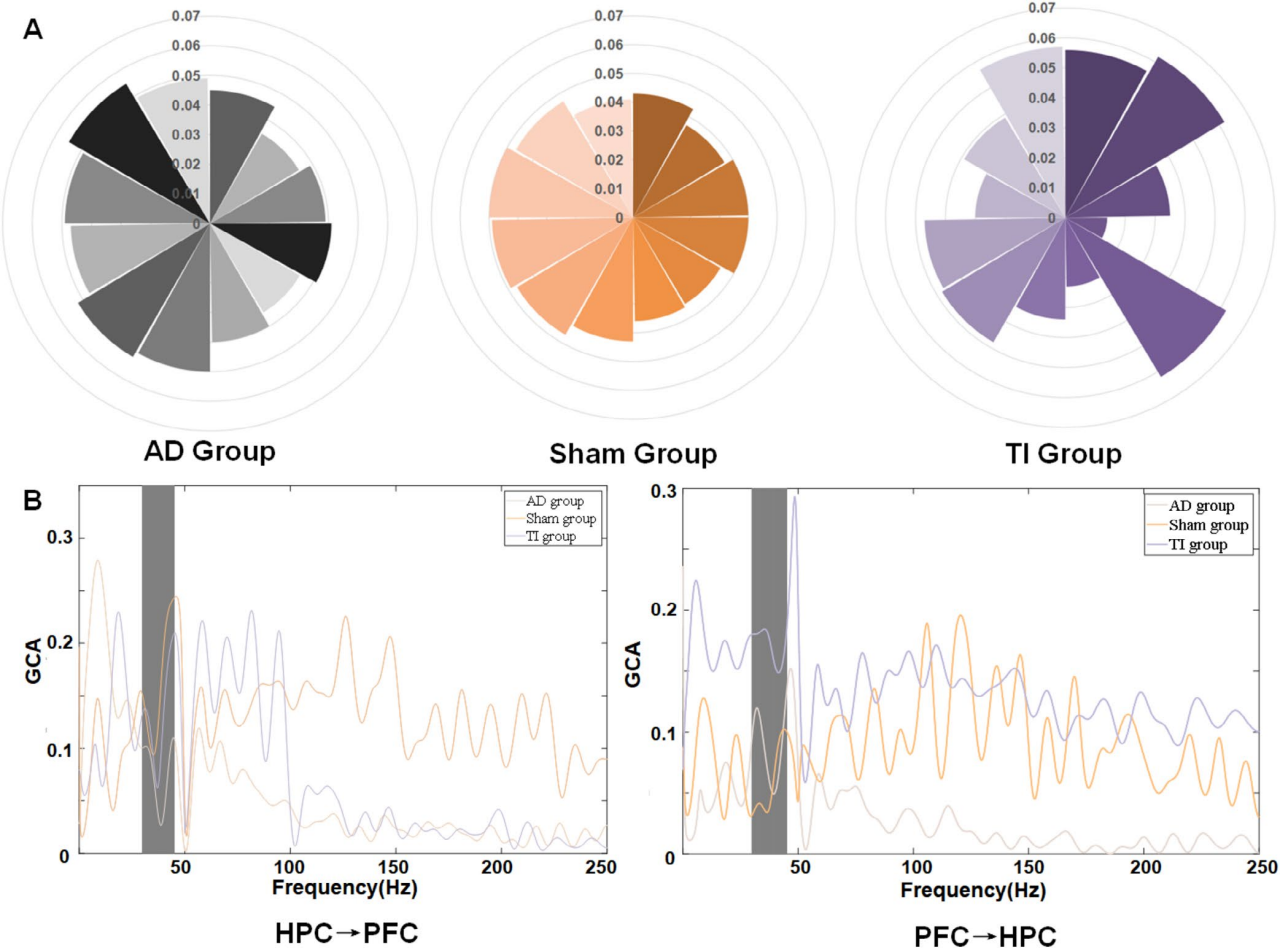
(A) Cross‐frequency coupling results across groups show that the TI group exhibits more dispersed coupling, indicating greater coupling variability, as illustrated by the higher values in Figure 2E. (B) Granger causality connectivity across groups depict the gamma frequency band (30–45 Hz) for analysis, considering the impact of power noise. The results indicate that the TI stimulation group shows significantly higher connectivity from PFC to HPC (*p* < 0.05), while connectivity from HPC to PFC does not differ significantly from other groups.

The LFP of the mice in the Y maze choice area (red circle) was next examined because it better captured the mice memory state at this stage of behavior, which was comparatively steady with little interference. Modifications in local field potentials brought on by stimulation in the theta and gamma frequency ranges were reflected in changes in behavioral performance. Gamma was our stimulation frequency band among them, and we found that the stimulated group of mice had significantly higher hippocampus gamma frequency band local field potentials (PSD_AD_ = 1.97e‐5 ± 0.92e‐5, PSD_Sham_ = 2.09e‐5 ± 0.88e‐5, PSD_TI_ = 4.81e‐5 ± 2.41e‐5, *p* < 0.05, Figure 2C). The theta frequency band, which was not stimulated, was next examined. We discovered that while the stimulated group of mice had higher local field potentials in the hippocampus theta frequency region, the differences were not statistically significant (PSD_AD_ = 2.52e‐4 ± 1.79e‐4, PSD_Sham_ = 2.34e‐4 ± 1.62e‐4, PSD_TI_ = 7.53e‐4 ± 7.56e‐4, p > 0.05, Figure 2D). We performed a joint analysis of the LFP in the theta and gamma frequency bands to better investigate the relationship between changes in theta frequency band LFP and gamma frequency band LFP. The results showed that TI stimulation increases theta‐gamma coupling (from 0.15 ± 0.25 to 0.26 ± 0.27), which was related to changes in theta band LFP (*p* = 0.041, Figures 2E and 3A). Using the prefrontal cortex (PFC) as an example (because it is part of the cortex), we examined the brain connectivity characteristics between the hippocampus (HPC) and other memory‐related areas of the brain due to the features of the brain network. Without substantially altering the connections leaving the hippocampus, we demonstrated that TI stimulation can improve the statistical predictive relationship of neural activity flowing from the prefrontal cortex to the hippocampus (as indicated by GCA) (from 0.08 ± 0.01 to 0.18 ± 0.02, *p* = 0.034, Figures 2F and 3B).

### 5.4 Gamma TI Stimulation Modulates the Expression of Excitatory and Inhibitory Neurotransmitters

**FIGURE 4:**
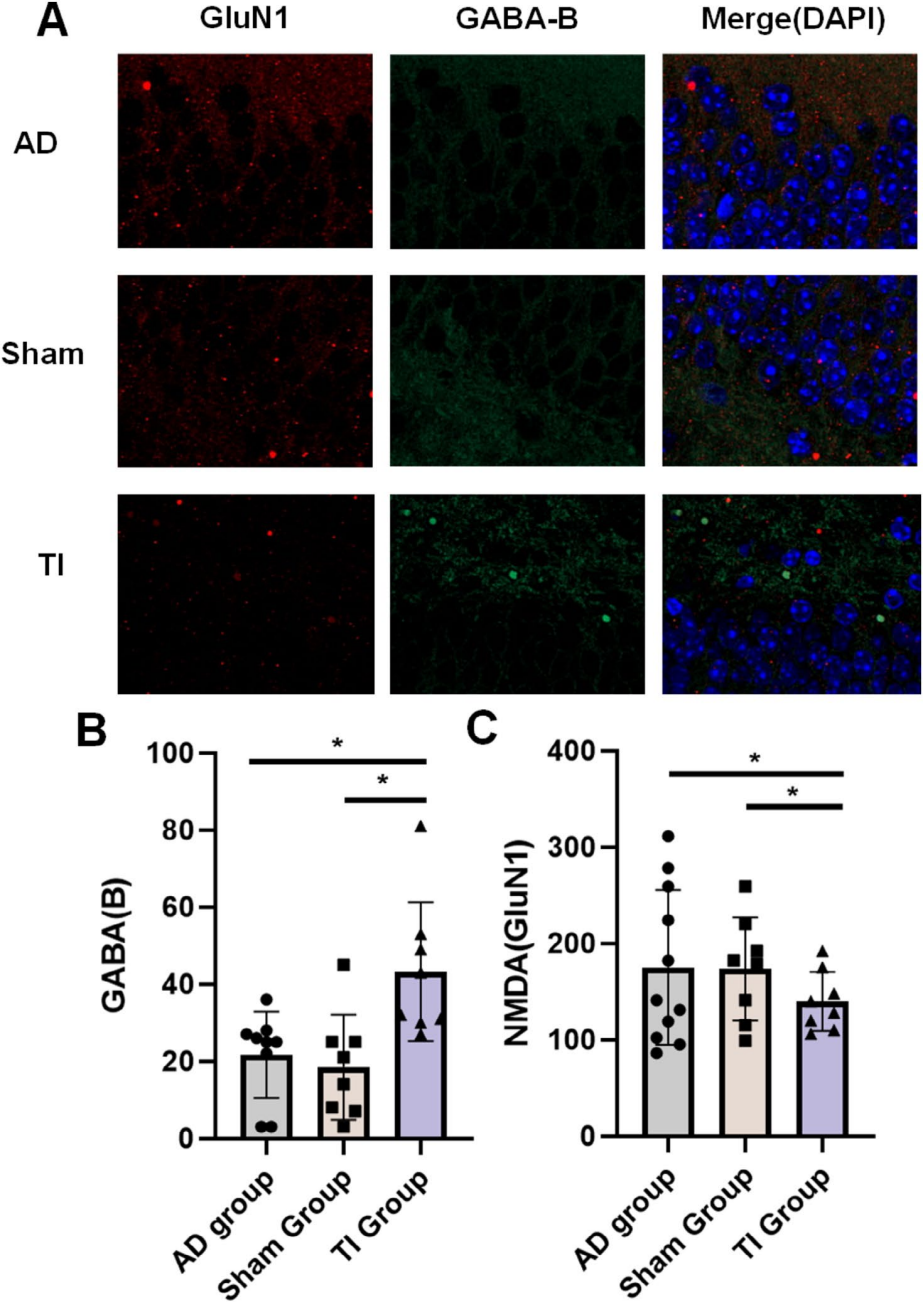
TI intervention influences the expression of excitatory neurotransmitter (GluN1) and inhibitory neurotransmitter (GABA‐B) (*p < 0.05). (A) Expression of neurotransmitters in the hippocampal target area of each AD mouse group. (B) Expression of GABA‐B. C. Expression of NMDA.

First, we confirmed the alterations in biochemical markers in the hippocampal regions of mice induced by TI. Following the 21‐day intervention, we assessed the GABA‐B and GluN1 release in each group of AD mice (Figure 4A). We discovered that TI 40 Hz stimulation reduced GluN1 receptor activity (decreased from 175.30 ± 80.30 to 139.90 ± 30.69, *p* = 0.047) and enhanced GABA‐B release (from 21.67 ± 11.25 to 43.25 ± 18.01, *p* = 0.031) in contrast to non‐40 Hz control stimulation (Figure 4B,C).

**FIGURE 5:**
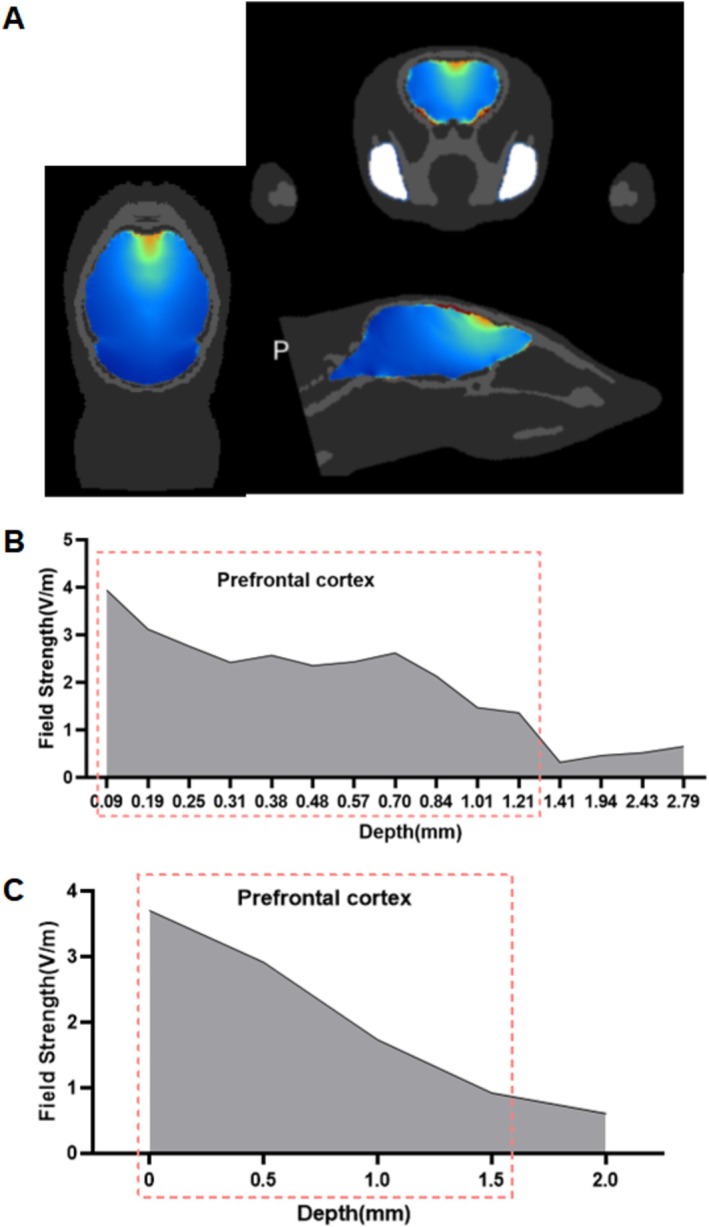
(A) Simulation diagram of the prefrontal cortex. (B) Simulation outcomes within the prefrontal cortex indicate that under these specified parameters, TI stimulation can focalize beyond 1.21 mm, with peak field strengths reaching up to 1 V/m. (C) Experimental findings in the prefrontal cortex confirm that TI stimulation achieves focalization in the frontal lobe, with peak field strengths reaching up to 1 V/m.

We compared the neurotransmitter staining results of TI stimulation in the hippocampus and TI stimulation in the prefrontal cortex based on our observation that TI influenced the granger causality connection between the hippocampus and the prefrontal cortex, and we performed both simulation and real measurements to ensure precise stimulation of the prefrontal cortex (Figure 5). This approach was consistent with the previous localization method for the hippocampus.

**FIGURE 6:**
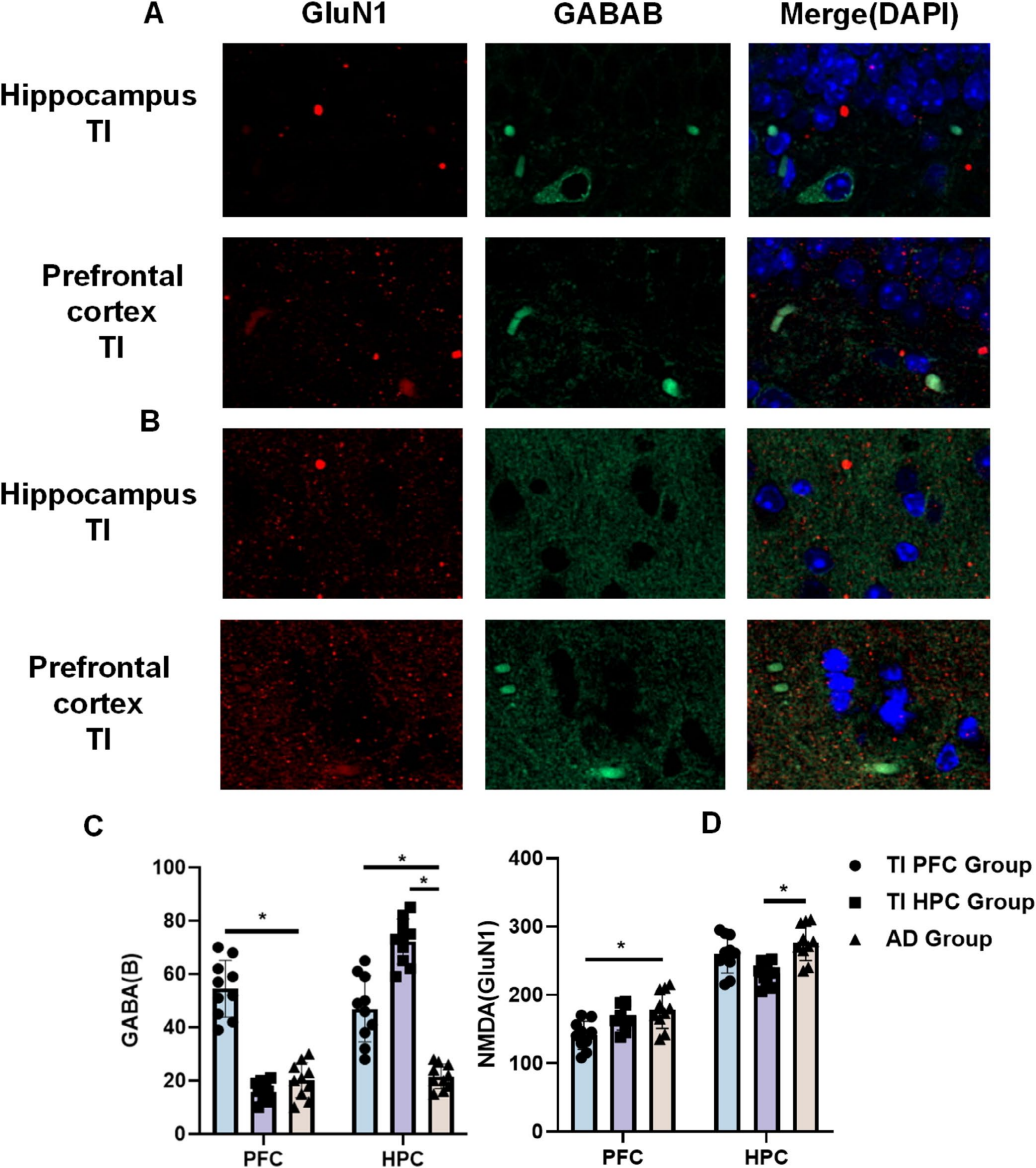
Expression of neurotransmitters in different target regions under stimulation (*p < 0.05). (A) Staining of hippocampus under two different stimulation. (B) Staining of prefrontal cortex under two different stimulation. (C) Expression of GABA‐B in the hippocampus and prefrontal cortex under different stimulation sites. (D) Expression of GluN1 in the hippocampus and prefrontal cortex under different stimulation sites.

To delve deeper into the modulatory effects of gamma stimulation on GABA and NMDA, we contrasted the stimulation effects on these neurotransmitters in different target areas. Staining of hippocampal and prefrontal regions in both stimulation groups revealed upregulation of GABA and downregulation of NMDA specifically at the stimulated region (Figure 6A,B). We observed no change in GABA levels in the prefrontal cortex of the hippocampal stimulation group, whereas significant differences in GABA expression were noted in the hippocampus of the prefrontal stimulation group (from 21.60 ± 4.57 to 46.90 ± 12.38, *p* = 0.045, Figure 6C). There were no significant differences observed both in the prefrontal cortex of the hippocampal stimulation group and the hippocampus of the prefrontal stimulation group (Figure 6D).

### 5.5 Gamma TI Stimulation Reduces Aβ Deposition in AD Mice

**FIGURE 7:**
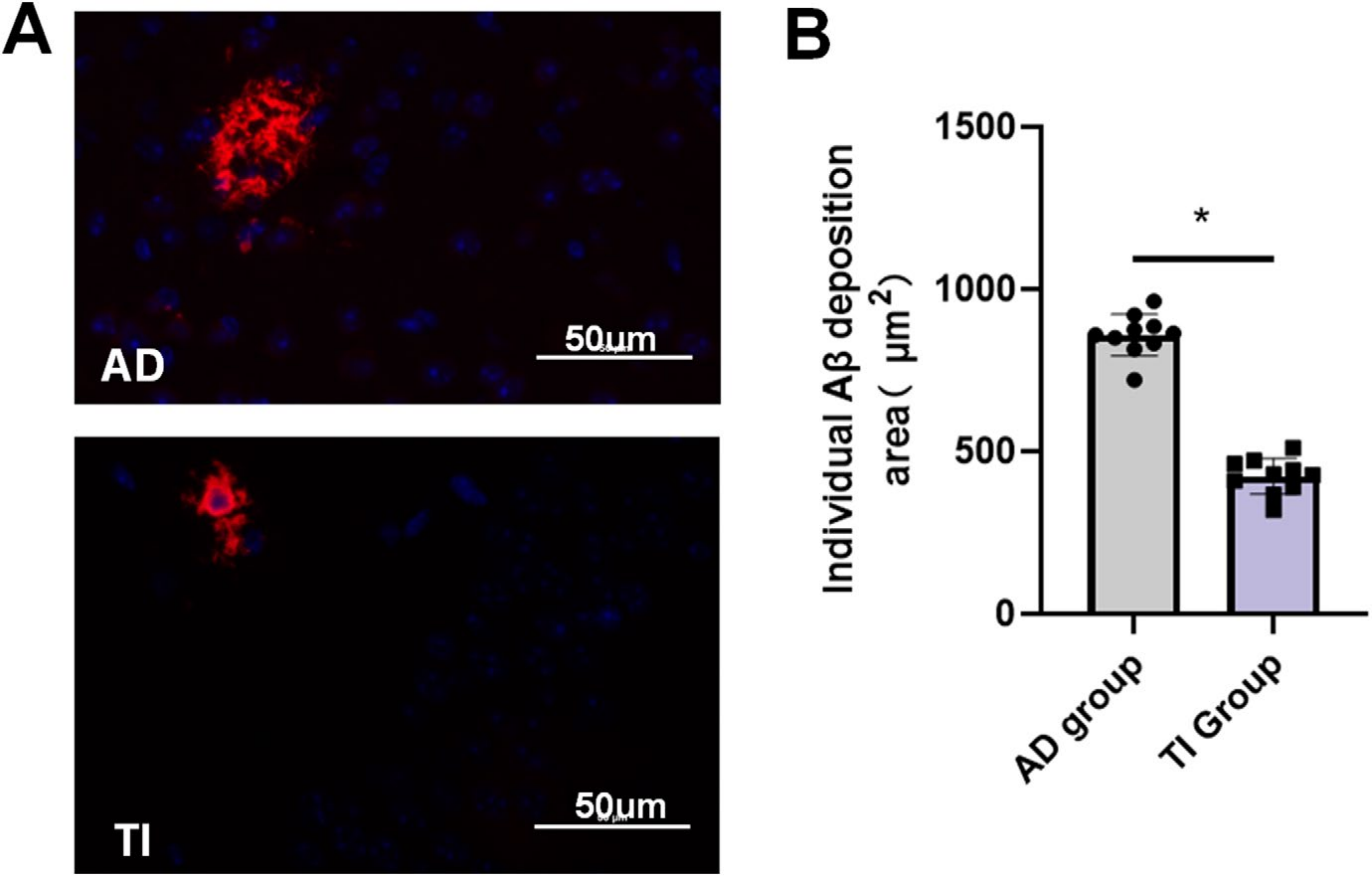
The impact of targeted transcranial inhibition (TI) on Aβ deposition in the hippocampus (*p < 0.05). A. Discrepancies in individual Aβ deposition areas at a scale of 50 μm before and after stimulation. B. Statistical analysis of individual Aβ deposition areas reveals that pre‐intervention, individual Aβ deposits measure 833 ± 97 μm^2^, while post‐intervention, this area decreases to 423 ± 76 μm^2^, with a significance level of *p* = 0.024.

According to studies, synaptic plasticity controlled by NMDA receptors may have an impact on the buildup of Aβ in Alzheimer's disease patients' brains, which may impair cognitive memory abilities [12]. Furthermore, excitotoxicity—which is linked to neuronal death in the brain afflicted by Alzheimer's disease—is linked to excessive NMDA receptor activation. Thus, after stimulation, we additionally examined Aβ deposition in the target area (the hippocampal region). With TI intervention, we saw a decrease in Aβ deposition in the area of the brain that was activated (decreased from 858.70 ± 64.51 to 423.4 ± 54.49, *p < 0.05*, Figure 7).

## 6 Discussion

This study investigated the mechanisms of TI stimulation by non‐invasively electrically stimulating Alzheimer's disease (AD) mice using TI kHz electric fields, with an emphasis on correlating physiological, molecular, and behavioral results to elucidate how targeted TI stimulation improves AD‐related deficits.

First, TI stimulation can specifically target the hippocampus while reducing activation of the overlaying cortex, as demonstrated by electric field modeling and in vivo measurements. The simulated maximum electric field amplitude in the hippocampus was 3.7 V/m. The actual in vivo measurements values were also significantly higher than in non‐target regions, which was consistent with previous findings that TI technology allows for non‐invasive modulation of deep brain structures [14]. The measured outermost field strength of the cortex is greater than the simulated results. Due to craniotomy surgery, the collecting electrodes on the surface of the cortex inevitably accumulate some stimulating currents, thereby increasing the field strength of the cortex. This further supports the accuracy of the measured data. This demonstrates that, during the experiment, the animals were stimulated by TI at the hippocampus region instead of transcranial alternating current stimulation. TI stimulation can specifically activate the hippocampus while minimizing cortical activation under certain circumstances as much as possible. This is also consistent with previous research findings [31].

Next, we determined through behavioral experiments that TI stimulation can improve the performance of Y maze tasks in AD mice, and linked behavioral improvements in AD mice to neurophysiological alterations brought on by TI by analyzing the electrophysiological data during the task process in mice. We speculate that the stimulation itself might promote inhibitory neurotransmitter release throughout the stimulation period, lowering excitatory neurotransmitter activity and reestablishing normal neuronal discharge in tandem with electrophysiological data. TI stimulation improved memory accuracy in Y‐maze tests (using parameters compatible with typical non‐invasive transcranial electrical stimulation (tES) protocols [32]), which is in line with more general findings that tES affects cognitive function [33]. In terms of neurophysiology, gamma‐frequency TI stimulation of the hippocampus restored Granger causality‐based functional connection between the hippocampus and prefrontal cortex in AD mice under task state, enhanced gamma‐band neuronal discharge, and controlled theta oscillations through cross‐frequency coupling. Consistent with previous studies, these results demonstrate that the activity of the right hippocampus is altered during the memory process [34]. Sprugnoli showed that 40 Hz tACS improves hippocampus function in moderate AD patients, which is in line with our finding that gamma‐frequency TI stimulation improves memory [54]. Memory functions are associated with cross‐frequency coupling itself [35]. Notably, the restoration of these gamma‐band oscillations and interregional linkages resulted in the greatest behavioral improvements, confirming the documented function of cross‐frequency coupling and hippocampus gamma synchrony in memory formation [36, 37]. By showing that TI stimulation can correct AD‐related abnormalities in hippocampus‐PFC connection, which are known to memory function, our findings build on recent research [33].

We examined TI‐induced alterations in GABA and NMDA expression and connected them with neurophysiological and behavioral results in order to deconstruct the molecular mechanisms behind these effects. In line with research that links a hippocampus GABA/NMDA imbalance to cognitive deficits in AD [38], we found that TI stimulation resulted in increased hippocampal GABA and decreased NMDA expression. Given that pyramidal cells are the primary source of gamma activity and that GABAergic interneurons modulate it, this neurotransmitter modulation most likely facilitates the restoration of gamma oscillations [39]. Our findings support those of Zhang et al., who found that gamma synchrony is disrupted by GABAergic interneuron loss in APP/PS1 mice [40]. Biochemical results indicate that the stimulation itself promotes the release of inhibitory neurotransmitters, reduces the activity of excitatory neurotransmitters, and reconstructs normal neuronal discharges in combination with electrophysiological data throughout the entire stimulation period. In addition, Aβ staining results indicate that TI stimulation also reduces Aβ deposition in the hippocampus. Combined with other staining results, we believe that this may be the result of enhanced neurotransmission (upregulation of GABA/downregulation of NMDA), This is consistent with Iaccarino et al., who showed that 40 Hz stimulation lowers Aβ [41]. To verify the focusing characteristics of TI stimulation and further validate the intervention mechanism of TI stimulation, we compared the HPC and PFC stimulation groups to ensure that these neurotransmitter alterations were caused by direct TI regulation of the hippocampus rather than indirect cortical effects. A precise TI intervention may selectively change neurotransmitter release at specific places, differentiating the immunofluorescence expression of prefrontal and hippocampal stimulation with respect to non‐stimulated areas, according to our hypothesis, given their potential link [42]. GABAergic activity was recorded from the prefrontal cortex to the hippocampus, indicating the interconnectedness of the brain, while NMDA transmission was not detected. Utilizing the PFC's dominant expression effect on hippocampus GABA (but not NMDA) expression [43, 44, 45], we discovered that: (1) hippocampus GABA and NMDA were changed by hippocampal stimulation (but not the PFC stimulation); and (2) PFC stimulation changed GABA/NMDA in the PFC and indirectly controlled hippocampus GABA (but not NMDA). These findings demonstrate that, in contrast to traditional tES (e.g., tACS), which frequently depends on non‐specific cortical activation, TI stimulation can directly regulates neurotransmitter release in target locations while minimizing the impact on nontarget areas [46].

This offers a mechanistic explanation for how TI's enveloping stimulation can control neurotransmitter release to enhance behavioral function and Aβ deposition, thereby improving cognitive function in AD mice. The results show that TI stimulation can precisely target the hippocampus and regulate neuronal firing in the hippocampus through GABA and NMDA, restoring its gamma oscillations to enhance memory function. Among our immunofluorescence and behavioral research limitations are their modest sample sizes, which are adequate to demonstrate physiological and behavioral effects but require validation with larger cohorts. Furthermore, our study was limited by its exclusive focus on neurotransmitters, which omitted the assessment of receptors such as GABA‐A and GluN2. Our non‐invasive brain stimulation by TI, however, is consistent with studies using DBS and neurotransmitter immunofluorescence, which at least indicate that GABA and NMDA are key biochemical indicators in the TI intervention mechanism [8, 47]. Overall, we saw good tolerance to TI stimulation at greater currents for the mouse experiment, with very minor side effects and no recorded adverse reactions. The current density under the parameter settings of this experiment is consistent with the current density used in tES research, and the current density is within the safe range for non‐invasive brain stimulation [48].

In conclusion, our results show that enhanced memory in AD mice, restored gamma oscillations/interregional connection, and TI‐induced hippocampal GABA/NMDA modulation are all related. A significant drawback of traditional neuromodulation techniques is addressed by TI, which targets deep brain areas non‐invasively. Our future research plans to continue examining the long‐term effects of TI stimulation, assess TI's translational potential in clinical AD populations, and refine electrode designs and current parameters to target more deep brain regions.

## Author Contributions

L.W., T.L., and J.W. designed the ferret experiments; L.W., L.H., T.L., L.L., and J.W. programmed the experiment; L.W., L.H., and S.L. acquired electrophysiological data for the experiment, L.W., P.Z., and Z.L. analyzed the data and edited the thoughts, and all authors wrote the manuscript.

## Funding

This work was supported by Shaanxi Province Key Research and Development Program Project (Grant 2025SF‐YBXM‐254), National Natural Science Foundation of China (Grant No. 82102179), Natural Science Foundation of Shandong Province (Grant Nos. ZR2024QF287, ZR2024YQ077), the Clinical Research Capacity Improvement Special Project of University of Health and Rehabilitation Sciences under Grant K2025LC0102, and the Taishan Scholar of Shandong Province (tsqn202211226 and tstp20221144). Scientific Research Innovation Capability Support Project for Young Faculty (SRICSPYF‐BS2025076).

## Ethics Statement

All procedures performed in studies involving mice were in accordance with the ethical standards of the institutional research committee. Ethics approval was obtained from the local Ethical Committee (Xi'an Jiaotong University's Animal Protection and Use Committee, Xi'an, China, Xijiao Life Sciences No. 2019‐36).

## Consent

The authors have nothing to report.

## Conflicts of Interest

The authors declare no conflicts of interest.

## Data Availability

Due to the large file size, the data sets generated and analyzed during the current study are available from the corresponding author upon request. Custom Matlab code that was used to analyze is available from the corresponding author upon request.

## References

[1] G. Buzsáki and A. Draguhn , “Neuronal Oscillations in Cortical Networks,” Science 304, no. 5679 (2004): 1926–1929, 10.1126/science.1099745. 15218136

[2] S. Hijazi , A. B. Smit , and R. E. van Kesteren , “Fast‐Spiking Parvalbumin‐Positive Interneurons in Brain Physiology and Alzheimer's Disease,” Molecular Psychiatry 28, no. 12 (2023): 4954–4967, 10.1038/s41380-023-02168-y. 37419975 PMC11041664

[3] P. J. Uhlhaas and W. Singer , “Neural Synchrony in Brain Disorders: Relevance for Cognitive Dysfunctions and Pathophysiology,” Neuron 52, no. 1 (2006): 155–168, 10.1016/j.neuron.2006.09.020. 17015233

[4] S. Gaubert , F. Raimondo , M. Houot , et al., “LFP Evidence of Compensatory Mechanisms in Preclinical Alzheimer's Disease,” Brain 142, no. 7 (2019): 2096–2112, 10.1093/brain/awz150. 31211359

[5] G. Sprugnoli , F. Munsch , D. Cappon , et al., “Impact of Multisession 40Hz tACS on Hippocampal Perfusion in Patients With Alzheimer's Disease,” Alzheimer's Research & Therapy 13, no. 1 (2021): 203, 10.1186/s13195-021-00922-4. PMC8690894 34930421

[6] F. Maestú , W. de Haan , M. A. Busche , and J. DeFelipe , “Neuronal Excitation/Inhibition Imbalance: Core Element of a Translational Perspective on Alzheimer Pathophysiology,” Ageing Research Reviews 69 (2021): 101372, 10.1016/j.arr.2021.101372. 34029743

[7] S. Jo , O. Yarishkin , Y. J. Hwang , et al., “GABA From Reactive Astrocytes Impairs Memory in Mouse Models of Alzheimer's Disease,” Nature Medicine 20, no. 8 (2014): 886–896, 10.1038/nm.3639. PMC8385452 24973918

[8] L. Yang , W. Liu , L. Shi , et al., “NMDA Receptor‐Arc Signaling Is Required for Memory Updating and Is Disrupted in Alzheimer's Disease,” Biological Psychiatry 94, no. 9 (2023): 706–720, 10.1016/j.biopsych.2023.02.008. 36796600 PMC10423741

[9] M. V. Fogaça , M. Wu , C. Li , X. Y. Li , M. R. Picciotto , and R. S. Duman , “Inhibition of GABA Interneurons in the mPFC Is Sufficient and Necessary for Rapid Antidepressant Responses,” Molecular Psychiatry 26, no. 7 (2021): 3277–3291, 10.1038/s41380-020-00916-y. 33070149 PMC8052382

[10] A. B. Ali , A. Islam , and A. Constanti , “The Fate of Interneurons, GABA_A_ Receptor Sub‐Types and Perineuronal Nets in Alzheimer's Disease,” Brain Pathology 33, no. 1 (2023): e13129, 10.1111/bpa.13129. 36409151 PMC9836378

[11] S. Li , G. Zhang , and J. Yang , “Role of NMDA Receptor‐Mediated Abnormalities of GABAergic Interneurons in Psychiatric Disorders,” Zhong Nan Da Xue Xue Bao. Yi Xue Ban 45, no. 2 (2020): 176–180, 10.11817/j.issn.1672-7347.2020.190048. 32386044

[12] R. Wang and P. H. Reddy , “Role of Glutamate and NMDA Receptors in Alzheimer's Disease,” Journal of Alzheimer's Disease 57, no. 4 (2017): 1041–1048, 10.3233/JAD-160763. PMC5791143 27662322

[13] A. Carles , A. Freyssin , F. Perin‐Dureau , G. Rubinstenn , and T. Maurice , “Targeting N‐Methyl‐d‐Aspartate Receptors in Neurodegenerative Diseases,” International Journal of Molecular Sciences 25, no. 7 (2024): 3733, 10.3390/ijms25073733. 38612544 PMC11011887

[14] S. Rampersad , B. Roig‐Solvas , M. Yarossi , et al., “Prospects for Transcranial Temporal Interference Stimulation in Humans: A Computational Study,” NeuroImage 202 (2019): 116124, 10.1016/j.neuroimage.2019.116124. 31473351 PMC6819277

[15] J. von Conta , F. H. Kasten , B. Ćurčić‐Blake , A. Aleman , A. Thielscher , and C. S. Herrmann , “Interindividual Variability of Electric Fields During Transcranial Temporal Interference Stimulation (tTIS),” Scientific Reports 11, no. 1 (2021): 20357, 10.1038/s41598-021-99749-0. 34645895 PMC8514596

[16] I. R. Violante , K. Alania , A. M. Cassarà , et al., “Non‐Invasive Temporal Interference Electrical Stimulation of the Human Hippocampus,” Nature Neuroscience 26, no. 11 (2023): 1994–2004, 10.1038/s41593-023-01456-8. 37857775 PMC10620081

[17] K. Alania , J. Borella , I. Violante , et al., “Non‐Invasive Temporal Interference Hippocampal Stimulation in Early Alzheimer's Disease,” Alzheimer's & Dementia 19 (2023): 21, 10.1002/alz.077433.

[18] F. Missey , M. S. Ejneby , I. Ngom , et al., “Obstructive Sleep Apnea Improves With Non‐Invasive Hypoglossal Nerve Stimulation Using Temporal Interference,” Bioelectronic Medicine 9, no. 1 (2023): 18, 10.1186/s42234-023-00120-7. 37553702 PMC10410873

[19] S. Ma , X. Song , T. Guo , et al., “Improving Spatial Resolution and Selectivity of Transcorneal Electrical Stimulation by Temporal Interference Technology,” Annu Int Conf IEEE Eng Med Biol Soc 2023 (2023): 1–4, 10.1109/EMBC40787.2023.10341049. 38083661

[20] B. Dogdas , D. Stout , A. F. Chatziioannou , and R. M. Leahy , “Digimouse: A 3D Whole Body Mouse Atlas From CT and Cryosection Data,” Physics in Medicine & Biology 52, no. 3 (2007): 577–587. 17228106 10.1088/0031-9155/52/3/003 PMC3006167

[21] A. Fabri , G.‐J. Giezeman , L. Kettner , S. Schirra , and S. Schönherr , “On the Design of CGAL a Computational Geometry Algorithms Library,” Software: Practice and Experience 30, no. 11 (2000): 1167–1202.

[22] P. Dular , C. Geuzaine , F. Henrotte , and W. Legros , “A General Environment for the Treatment of Discrete Problems and Its Application to the Finite Element Method,” IEEE Transactions on Magnetics 34, no. 5 (1998): 3395–3398.

[23] O. A. Shipton , M. El‐Gaby , J. Apergis‐Schoute , et al., “Left‐Right Dissociation of Hippocampal Memory Processes in Mice,” Proceedings of the National Academy of Sciences of the United States of America 111, no. 42 (2014): 15238–15243, 10.1073/pnas.1405648111. 25246561 PMC4210314

[24] ZMT , “Sim4Life,” [Online], https://www.zmt.swiss.

[25] L. Wu , W. Zhang , S. Li , et al., “Transcranial Alternating Current Stimulation Improves the Memory Function of Alzheimer's Mice by Ameliorating Abnormal Gamma Oscillation,” IEEE Transactions on Neural Systems and Rehabilitation Eegineering 31 (2023): 2060–2068. 10.1109/TNSRE.2023.3265378 37079421

[26] M. Hajos , A. Boasso , A. Cimenser , et al., “Sensory‐Evoked 40Hz Gamma Oscillation: A Feasible and Promising Treatment Option for Alzheimer's Disease,” Alzheimer's & Dementia 19, no. Sup16 (2023): 2, 10.1002/alz.072957.

[27] M. Cleal , B. D. Fontana , D. C. Ranson , et al., “The Free‐Movement Pattern Y‐Maze: A Cross‐Species Measure of Working Memory and Executive Function,” Behavior Research Methods 53, no. 2 (2021): 536–557, 10.3758/s13428-020-01452-x. 32748238 PMC8062322

[28] C. Kong , J. W. Ahn , S. Kim , et al., “Long‐Lasting Restoration of Memory Function and Hippocampal Synaptic Plasticity by Focused Ultrasound in Alzheimer's Disease,” Brain Stimulation 16, no. 3 (2023): 857–866, 10.1016/j.brs.2023.05.014. 37211337

[29] M. S. Treder , I. Charest , S. Michelmann , et al., “The Hippocampus as the Switchboard Between Perception and Memory,” Proceedings of the National Academy of Sciences of the United States of America 118, no. 50 (2021): e2114171118, 10.1073/pnas.2114171118. 34880133 PMC8685930

[30] S. Song , J. Zhang , Y. Tian , et al., “Temporal Interference Stimulation Regulates Eye Movements and Neural Activity in the Mice Superior Colliculus,” Annual International Conference of EMBC (2021): 6231–6234, 10.1109/EMBC46164.2021.9629968. 34892538

[31] M. Vöröslakos , Y. Takeuchi , K. Brinyiczki , et al., “Direct Effects of Transcranial Electric Stimulation on Brain Circuits in Rats and Humans,” Nature Communications 9, no. 1 (2018): 483, 10.1038/s41467-018-02928-3. PMC5797140 29396478

[32] L. Wu , W. Zhang , S. Li , et al., “Transcranial Alternating Current Stimulation Improves Memory Function in Alzheimer's Mice by Ameliorating Abnormal Gamma Oscillation,” IEEE Transactions on Neural Systems and Rehabilitation Engineering 31 (2023): 2060–2068, 10.1109/TNSRE.2023.3265378. 37079421

[33] N. R. Nissim , D. V. H. Pham , T. Poddar , E. Blutt , and R. H. Hamilton , “The Impact of Gamma Transcranial Alternating Current Stimulation (tACS) on Cognitive and Memory Processes in Patients With Mild Cognitive Impairment or Alzheimer's Disease: A Literature Review,” Brain Stimulation 16, no. 3 (2023): 748–755, 10.1016/j.brs.2023.04.001. 37028756 PMC10862495

[34] Y. Sakaguchi and Y. Sakurai , “Left‐Right Functional Difference of the Rat Dorsal Hippocampus for Short‐Term Memory and Long‐Term Memory,” Behavioural Brain Research 382 (2020): 112478, 10.1016/j.bbr.2020.112478. 31935420

[35] R. T. Canolty and R. T. Knight , “The Functional Role of Cross‐Frequency Coupling,” Trends in Cognitive Sciences 14, no. 11 (2010): 506–515, 10.1016/j.tics.2010.09.001. 20932795 PMC3359652

[36] R. Scheffer‐Teixeira and A. B. Tort , “On Cross‐Frequency Phase‐Phase Coupling Between Theta and Gamma Oscillations in the Hippocampus,” eLife 5 (2016): e20515, 10.7554/eLife.20515. 27925581 PMC5199196

[37] M. A. Belluscio , K. Mizuseki , R. Schmidt , R. Kempter , and G. Buzsáki , “Cross‐Frequency Phase‐Phase Coupling Between θ and γ Oscillations in the Hippocampus,” Journal of Neuroscience 32, no. 2 (2012): 423–435, 10.1523/JNEUROSCI.4122-11.2012. 22238079 PMC3293373

[38] S. Gunasekaran and R. V. Omkumar , “miR‐146a and miR‐200b Alter Cognition by Targeting NMDA Receptor Subunits,” iScience 25, no. 12 (2022): 105515, 10.1016/j.isci.2022.105515. 36561887 PMC9763852

[39] L. Verret , E. O. Mann , G. B. Hang , et al., “Inhibitory Interneuron Deficit Links Altered Network Activity and Cognitive Dysfunction in Alzheimer Model,” Cell 149, no. 3 (2012): 708–721, 10.1016/j.cell.2012.02.046. 22541439 PMC3375906

[40] M. Chen , Y. Chen , Q. Huo , et al., “Enhancing GABAergic Signaling Ameliorates Aberrant Gamma Oscillations of Olfactory Bulb in AD Mouse Models,” Molecular Neurodegeneration 16, no. 1 (2021): 14, 10.1186/s13024-021-00434-7. 33663578 PMC7934466

[41] H. F. Iaccarino , A. C. Singer , A. J. Martorell , et al., “Gamma Frequency Entrainment Attenuates Amyloid Load and Modifies Microglia,” Nature 540 (2016): 230–235, 10.1038/nature20587. 27929004 PMC5656389

[42] G. A. Czapski and J. B. Strosznajder , “Glutamate and GABA in Microglia‐Neuron Cross‐Talk in Alzheimer's Disease,” International Journal of Molecular Sciences 22, no. 21 (2021): 11677, 10.3390/ijms222111677. 34769106 PMC8584169

[43] J. A. McQuail , C. Bañuelos , C. L. LaSarge , M. M. Nicolle , and J. L. Bizon , “GABA(B) Receptor GTP‐Binding Is Decreased in the Prefrontal Cortex but Not the Hippocampus of Aged Rats,” Neurobiology of Aging 33, no. 6 (2012): 1124.e1–1124.e12, 10.1016/j.neurobiolaging.2011.11.011. PMC3310948 22169202

[44] R. Malik , Y. Li , S. Schamiloglu , and V. S. Sohal , “Top‐Down Control of Hippocampal Signal‐To‐Noise by Prefrontal Long‐Range Inhibition,” Cell 185, no. 9 (2022): 1602–1617.e17, 10.1016/j.cell.2022.04.001. 35487191 PMC10027400

[45] S. R. Tanqueiro , F. M. Mouro , C. B. Ferreira , et al., “Sustained NMDA Receptor Hypofunction Impairs Brain‐Derived Neurotropic Factor Signalling in the PFC, but Not in the Hippocampus, and Disturbs PFC‐Dependent Cognition in Mice,” Journal of Psychopharmacology 35, no. 6 (2021): 730–743, 10.1177/02698811211008560. 34008450

[46] M. Wischnewski , M. Engelhardt , M. A. Salehinejad , D. J. L. G. Schutter , M. F. Kuo , and M. A. Nitsche , “NMDA Receptor‐Mediated Motor Cortex Plasticity After 20 Hz Transcranial Alternating Current Stimulation,” Cerebral Cortex 29, no. 7 (2019): 2924–2931, 10.1093/cercor/bhy160. 29992259

[47] G. Carello‐Collar , B. Bellaver , P. C. L. Ferreira , et al., “The GABAergic System in Alzheimer's Disease: A Systematic Review With Meta‐Analysis,” Molecular Psychiatry 28, no. 12 (2023): 5025–5036, 10.1038/s41380-023-02140-w. 37419974

[48] D. A. Turner , S. Degan , F. Galeffi , S. Schmidt , and A. V. Peterchev , “Rapid, Dose‐Dependent Enhancement of Cerebral Blood Flow by Transcranial AC Stimulation in Mouse,” Brain Stimulation 14, no. 1 (2021): 80–87. 33217607 10.1016/j.brs.2020.11.012 PMC7855527

